# Five ways to improve international comparisons of cancer survival: lessons learned from ICBP SURVMARK-2

**DOI:** 10.1038/s41416-022-01701-0

**Published:** 2022-01-20

**Authors:** Therese M.-L. Andersson, Tor Åge Myklebust, Mark J. Rutherford, Bjørn Møller, Melina Arnold, Isabelle Soerjomataram, Freddie Bray, D. Maxwell Parkin, Paul C. Lambert

**Affiliations:** 1 Department of Medical Epidemiology and Biostatistics, Karolinska Institutet, Stockholm, Sweden; 2 Cancer Registry of Norway, Oslo, Norway; 3 Department of Research and Innovation, Møre and Romsdal Hospital Trust, Ålesund, Norway; 4 Biostatistics Research Group, Department of Health Sciences, University of Leicester, Leicester, UK; 5 Cancer Surveillance Branch, International Agency for Research on Cancer (IARC/WHO), Lyon, France; 6 School of Cancer & Pharmaceutical Sciences, King’s College London, London, UK; 7 INCTR Challenge Fund, Prama House, Oxford, UK

**Keywords:** Cancer epidemiology, Cancer epidemiology, Epidemiology

## Abstract

**Background:**

Comparisons of population-based cancer survival between countries are important to benchmark the overall effectiveness of cancer management. The International Cancer Benchmarking Partnership (ICBP) Survmark-2 study aims to compare survival in seven high-income countries across eight cancer sites and explore reasons for the observed differences. A critical aspect in ensuring comparability in the reported survival estimates are similarities in practice across cancer registries. While ICBP Survmark-2 has shown these differences are unlikely to explain the observed differences in cancer-specific survival between countries, it is important to keep in mind potential biases linked to registry practice and understand their likely impact.

**Methods:**

Based on experiences gained within ICBP Survmark-2, we have developed a set of recommendations that seek to optimally harmonise cancer registry datasets to improve future benchmarking exercises.

**Results:**

Our recommendations stem from considering the impact on cancer survival estimates in five key areas: (1) the completeness of the registry and the availability of registration sources; (2) the inclusion of death certification as a source of identifying cases; (3) the specification of the date of incidence; (4) the approach to handling multiple primary tumours and (5) the quality of linkage of cases to the deaths register.

**Conclusion:**

These recommendations seek to improve comparability whilst maintaining the opportunity to understand and act upon international variations in outcomes among cancer patients.

## Introduction

Comparisons of cancer survival between or within countries, as derived from population-based cancer registry data, have been the subject of a number of large-scale and influential collaborative projects, including the International Cancer Benchmarking Partnership (ICBP) [1**–**4]. As part of phase two of ICBP, Survmark-2 has sought to benchmark survival for eight cancer sites across 21 jurisdictions in seven countries, bringing together cancer clinicians, policy-makers, researchers and data experts [4, 5]. As well as documenting and elucidating cancer-specific survival differences [6**–**9], one of the aims of Survmark-2 is to investigate if they might, in part, be explained by differences in cancer registration methodology [10**–**12].

Most previous studies have concluded that, although differences in cancer registration practice could partially explain survival differences, they are unlikely to explain the large variations seen across countries [10, 12–16], as long as the data are not analysed too prematurely when both incident cases and links to death data might be lacking to a great extent. A number of international guidelines have been developed to ensure that the cancer registry data are collected, coded, processed and presented in a comparable way [17**–**23]. There are, however, some differences in their interpretation and implementation between registries, as well as variations in the sources (of data) available to registries. Even though differences in registration practice are unlikely to explain large differences in survival, documenting what the differences are, understanding how they impact on survival comparisons, and how such sources of variation can be minimised could be of benefit to registries and to researchers seeking to examine international survival comparisons. In this paper, we therefore draw upon some of the lessons learned from the ICBP Survmark-2 study with respect to registry practices and offer five recommendations that seek to increase data harmonisation so as to improve the comparability of future studies of cancer survival [1**–**3].

### Data sources and the potential for missed cases

Cancer registries commonly receive information on new cancer cases from multiple sources, the two most common being pathology reports and hospital admission data. The completeness, i.e. the extent to which all incident cancers occurring in the population are included [18], can vary by source, and the average number of sources per case registered is a useful indicator of completeness [18]. It is however equally important to distinguish between the sources and procedures used to identify new cases, and the sources which are subsequently used to find additional information about registered cases. Even though no cancer registry will capture all cancer cases, survival comparisons rely not only on the level of completeness, but whether those cases captured are truly representative. Cancer registries that rely heavily on pathology reports might miss cases that are not sent for a biopsy, for instance due to cases being of older age or having advanced stage (with a poorer prognosis and shorter survival time). In such circumstances, population-based survival estimates will be overestimated. On the other hand, cancer registries that rely more heavily on hospital admissions might instead miss cases with a better prognosis, e.g. those that are only treated in an outpatient setting, leading to cancer survival being underestimated. Thus while knowledge of sources used to identify new cancer cases is important, an understanding of the completeness of each source and the proportion of cases notified from each source is critical. Depending on the sources used for registering a new cancer case, registries might also be more or less likely to record a prevalent cancer of an individual that moves into the catchment area and has previously been diagnosed in another region, leading to duplicate records of the same individual but in different regions [24].

Capture–recapture methods can be used to investigate whether the proportion of cases notified by different sources varies according to registry variables [18, 25]. However, a drawback of the method is that it can not be used to estimate the number of cases that were not notified by one or more of the notification sources investigated [26]. Cancer registries should therefore not only describe the underlying processes, but record the specific source used in registering a new case or in augmenting information to an existing case. An understanding of which cases might be missed is also important in reporting survival comparisons across countries or over time.

### Use of death certificate to identify cases

Many cancer registries receive information on deaths in their target population for which “cancer” is mentioned on the death certificate, so-called death certificate notified (DCN) cases [19]. Cancer registries typically check DCN cases against their registered cases to see if this is a case already notified to the registry through other sources. Depending on the methodology of the registry (e.g. the sources of information available to the registry, and the delay with which information from each source arrives), comparing deaths with the file of registered cases may require a “waiting period” to be established, to ensure that all source reports are duly received. The length of the waiting period prior to checking DCN cases against the registry file will differ between registries, depending on how often and quickly information from other sources is retrieved. It should however be of sufficient length to ensure receipt of the information on cases from all other sources.

For the DCN cases for which there is no other source in the register following the waiting period, a traceback procedure is initiated to verify the diagnosis and that the date of diagnosis precedes the date of death. The cases for which no dates of diagnosis are found are referred to as death certificate only (DCO) cases, and the DCO cases together with the cases for which extra information are found are referred to as death certificate initiated (DCI) cases; those that would not be in the register if it was not for the notification from the death certificate. The proportion of DCO cases is often used as a quality measure of a cancer registry, however, the proportion of DCI is rarely reported. In most registries, the proportion of DCI is usually not known. As a measure of quality, the proportion of DCI is much more informative than the corresponding DCO proportion, since the DCI gives more information as to how many cancer cases are missed through routine registration processes. By itself, the DCO% is not an indicator of completeness of registration, a low DCO% may indicate efficient case-finding, but it could equally well result from the efficient traceback of DCN cases (although since the DCI% will always be equal to, or greater than, the DCO%, an elevated DCO% is suggestive of incompleteness) [18].

DCO cases are often excluded from survival analysis, since these cases do not have any follow-up information, the underlying reason why registries attempt to minimise the DCO%. However, little consideration is given to the fact that inclusion of the DCI cases with positive follow-up time will lead to a downward bias in the survival estimates [11]. This is because the DCI cases are a selective group of missed cases, that is, they are missed cases that die due to cancer, whereas the missed cases that are still alive or do not have cancer mentioned on their death certificates are still missed. Thus when comparing survival between registries with proportions of DCOs that are similar, comparisons are still biased if the proportion of DCIs are not. A registry that initially missed a large proportion of cases, but had very effective traceback and a registry that missed only a small fraction of cases with no traceback could have very similar proportion of DCO cases, even though their DCI proportion differ greatly. Another suggested bias resulting from inclusion of DCI cases is that the traceback procedures might not find the true date of diagnosis [27], although studies have shown that this can account for only a small part of the observed differences in survival estimates [12, 14, 16]. Differences in the amount of time and resources spent on traceback in establishing the date of diagnosis may lead to differences in accuracy and completeness between registries.

Simulation studies investigating errors introduced by DCI cases and incompleteness have shown however that any bias is likely to be small [12, 13, 16]. Even so, information on both the DCI and the DCO proportions are important when comparing survival across registries. Moreover, information on which cases are DCI cases can be informative in estimating completeness and in performing sensitivity analyses, and thus a key recommendation is that DCI cases are flagged in the registry database.

### Date of incidence

Since cancer patient survival is estimated based on the time between the date of incidence (DOI) and date of death; the DOI recorded at the registry evidently has some influence on survival estimates. In order to ensure consistency between registries in how they determine DOI, a hierarchical set of rules have been established, prioritising which source of information from which to define DOI. There are several available: the European Network of Cancer Registries (ENCR) rules [20] the International Association of Cancer Registries (IACR) and International Agency for Research on Cancer (IARC) rules [17] and the Surveillance, Epidemiology and End Results (SEER) rules [22]. The main difference between these rules is the priority given to different dates in selecting “date of incidence”. The IARC/IACR rules prioritise the first consultation or admission to a clinic or hospital for the malignancy in question, while the ENCR rules prioritise the date of histological or cytological confirmation. In a registry that uses the ENCR rules for instance, the DOI of a proportion of cases will be based on date of hospitalisation in the absence of morphological information. Even in registries using the same rules, there may be differences in the proportion of cases for which different dates are used, dependant on the information available. It is therefore recommended that registries record dates relevant to each information source—for example, date of (first) hospital admission, date of the pathology report, date of CT or MRI scan, date of first treatment—so that DOI can be standardised between the datasets being compared. In closing; however, we note that while DOI selection is often thought to be a problem in comparing survival, it has been shown to have a rather trivial influence, except on very short-term survival probabilities [10].

### Multiple primaries

Differences in the definition of multiple primaries have been discussed as a potential problem in assuring comparability of cancer patient survival estimates across countries [28]. Multiple primaries are primary tumours occurring in individuals who have been previously diagnosed with cancer, not extensions, recurrences or metastases. Such information on subsequent cancers, in the same or a different organ, can be important to record when investigating specific research questions, for example, studies on contralateral breast cancer or solid tumours following a diagnosis of a haematological malignancy [29]. Several sets of rules define what constitutes a new reportable cancer [21, 22]. The rules developed jointly by IACR and IARC are commonly used internationally, while those of the SEER programme are used mainly by North American registries. However, when estimating population-based cancer survival for a specific cancer type, only the first diagnosis of that specific type for each individual should be included in the analysis; individuals who have previously had cancer at a different site should be included. And if an individual is later diagnosed with another cancer at the same site this new cancer diagnosis should not be included in the survival analysis, and the follow-up for this individual should be continued. For some sites, there could be multiple primaries recorded for an individual on the same date, which could in some situations lead to differences between registries in which type of cancers are included in survival analysis.

Hence, the definition of multiple primaries used by the registry has limited impact on survival estimates, since the definition of what is a subsequent primary does not alter the information for the first diagnosis. However, in a recently-established registry there will be cases recorded as first cancers in individuals who had previous primary malignancies not recorded as they occurred in previous years prior to the beginning of registration. This may lead to the inclusion of second cancer at the same site that would not have been included if first cancer had been known. Since the prognosis is usually worse for recurring cancers and second primary cancers, this could lead to differences in survival estimates between registries of differing maturity. However, multiple cancers of the same site are rare (according to the international rules, they are only reportable if they are of a different histological type [21]). As long as only multiple primaries of the same site are excluded in survival analyses, and not any multiple primaries, this will have little impact on the estimates.

### Linking to mortality and vital statistics

In order to accurately estimate survival, the vital status of all cancer patients under study is required. In order to retrieve this information, many cancer registries link the cancer cases in the register to information from death registers or vital statistics offices. Most registries that use this approach to retrieve follow-up information assume that cases with no match are still alive, a situation that is often referred to as passive follow-up. If not all deaths are captured cancer survival will be overestimated. This is more likely a bigger problem for registries that do not have a unique identification or health care number for each individual, or where such a unique identifier is not always used, and the linkage therefore relies on matching on name, birth date, address, or other personal attributes based on probability matching. Another source of bias occurs when the registry does not capture vital status information on individuals that have emigrated from the country or moved out of the registry capture area, since these individuals are assumed alive, again leading to an overestimation of survival.

The problem with linking of death information and lacking emigration information leads to “immortals”: individuals for whom death information will never be retrieved and they appear in the register to live forever. Immortals are likely to have a larger impact on long-term survival than short-term survival, since problems with linkages gets larger over time as individuals change address or name, and a proportion of those still alive move out of the jurisdiction. It is still unclear as to the extent of this problem in international comparisons of survival. Registries can evaluate the potential for deaths to be missed, particularly when a country has a number of subnational registries. We recommend that registries review a random sample of long-term survivors, especially for cancers with poor prognosis, to try to establish if the vital status is correct. A review of all individuals alive beyond the age of 100 can also be done, and some registries do this as part of annual quality checks. Another indirect method to assess the accuracy of follow-up information is to estimate relative survival conditional on survival up to a number of years based on the number of years after diagnosis that statistical “cure” occurs for cancer under study. If the conditional survival is greater than 1, surviving cancer patients may have a better prognosis than would be expected, which could be due to missing linkages to deaths information.

## Discussion

International population-based cancer survival comparisons are important to benchmark the overall effectiveness of cancer management across countries. As differences in registration practice may impact on survival estimates and survival comparisons, registries should describe the specific situation with respect to the five areas of registry practice outlined herein studies to inform the results presented. Based on the ICBP SURVMARK-2 benchmarking studies [10] investigating the impact of these differences, they are only likely to explain a part of the observed survival differences. Even so, it is important to keep in mind potential biases based on registry practice, and understand how differences between registries likely impact on survival estimates. Standards and guidelines help to minimise the differences but factors such as legal aspects, national infrastructure or resources, e.g. for traceback, will lead to differences in registration practice. Since many sources of bias are due to missing information, such as cases not reported or cases assumed alive due to missing deaths information, it is not possible to reliably adjust or correct the data for the registration differences. Instead, one should try to understand how each issue dealt with in each registry, and undertake sensitivity analyses where possible.

**Fig. 1:**
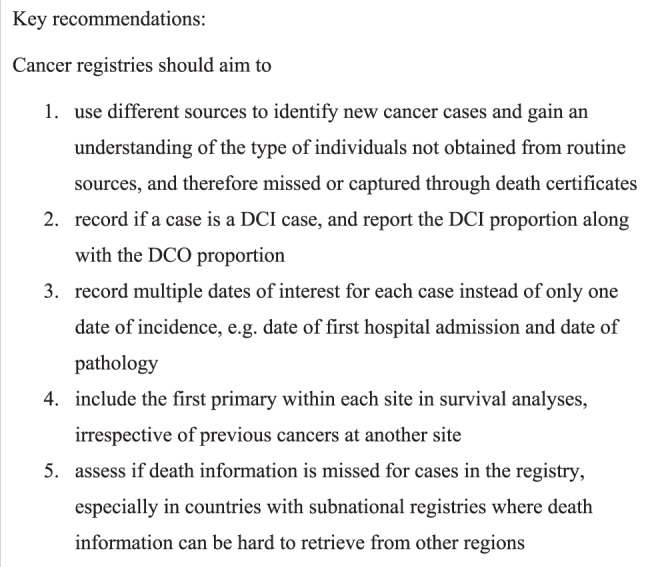
Key recommendations to cancer registries to improve cancer survival benchmarking. Based on lessons learned from ICBP SURVMARK-2.

Benchmarking cancer survival has been a driver for change in many individual jurisdictions; informing cancer service planning and policy leading to survival improvements over time. We have made some recommendations based on the experience of international survival comparison studies that seek to improve the quality of future benchmarking iterations, whilst also improving the validity of internal cancer survival estimates in each jurisdiction. A summary of the recommendations can be found in Fig. 1. These recommendations will improve comparability whilst maintaining the opportunity to understand and act upon international variations in outcomes among cancer patients.

## Data Availability

Not applicable.
